# Comparative efficacy of a secretory phospholipase A_2 _inhibitor with conventional anti-inflammatory agents in a rat model of antigen-induced arthritis

**DOI:** 10.1186/ar3278

**Published:** 2011-03-14

**Authors:** Liam G Coulthard, Jaclyn Costello, Brent Robinson, Ian A Shiels, Stephen M Taylor, Trent M Woodruff

**Affiliations:** 1 School of Biomedical Sciences, Research Road, University of Queensland, St. Lucia, Queensland, 4072, Australia

## Abstract

**Introduction:**

Previously, secretory phospholipase A_2 _(sPLA_2_) inhibition has been used as an adjunct to conventional rheumatoid arthritis therapy in human clinical trials without significant improvement of arthritic pathology. In this study, we compared the efficacy of a potent and orally active group IIa secretory phospholipase A_2 _inhibitor (sPLA_2_I) to conventional anti-arthritic agents; infliximab, leflunomide and prednisolone, in a rat model of antigen-induced arthritis.

**Methods:**

Initially, to establish efficacy and dose-response, rats were orally dosed with the sPLA_2_I (1 and 5 mg/kg) two days prior to arthritis induction, and then daily throughout the 14-day study period. In the second trial, rats were orally dosed with the sPLA_2_I (5 and 10 mg/kg/day) beginning two days after the induction of arthritis, at the peak of joint swelling. Separate groups of rats were also dosed with the tumour necrosis factor-alpha (TNF-α) inhibitor infliximab (single 3 mg/kg i.v. injection), leflunomide (10 mg/kg/day, oral) or prednisolone (1 mg/kg/day, oral) at this same time point and used as comparative treatments.

**Results:**

In the pathology prevention trial, both 1 and 5 mg/kg dose groups of sPLA_2_I demonstrated a significant reduction in joint swelling and gait disturbances; however, only the higher 5 mg/kg dose resulted in significantly reduced histopathology scores. In the post-induction trial, rats dosed with sPLA_2_I showed a significant improvement in joint swelling and gait scoring, whereas none of the conventional therapeutics achieved a significant decrease in both of these two disease markers. Histopathological scoring at the end-point of the study demonstrated significantly reduced median scores in rats treated with 10 mg/kg sPLA_2_I and leflunomide.

**Conclusions:**

The results from this study suggest a pathogenic role for sPLA_2 _enzymes in this model of arthritis in rats, and the potential clinical utility of sPLA_2 _inhibition as a safer, and more effective, alternative to conventional anti-arthritic therapeutics.

## Introduction

Rheumatoid arthritis (RA) is an immune-based chronic inflammatory synovitis presenting with pain, stiffness and swelling of the affected joints. RA results in secondary bone and cartilage destruction causing joint deformity. Current therapies include conventional non-steroidal anti-inflammatory agents (NSAIDs), corticosteroids such as prednisolone, disease-modifying anti-rheumatic-drugs, such as methotrexate or leflunomide, and biological therapies such as the inhibitors of tumour necrosis factor alpha (TNFα), etanercept, adulimumab and infliximab [1]. No single agent is completely effective at treating disease pathology and is devoid of side effects; consequently, a safe and effective treatment for RA remains elusive. In the mid-1980's, phospholipase A,_2 _(PLA_2_) enzymes were found to be highly expressed in the synovial fluid of RA patients [2]. PLA_2 _forms a group of enzymes that metabolise phosphoglycerides to release lipid mediators such as lysophospholipids and arachidonic acid. These metabolites can be converted into the pro-inflammatory platelet activating factor (PAF) and eicosanoids (prostaglandins, thromboxanes, and leukotrienes), respectively [3]. As opposed to cytosolic PLA_2 _enzymes which have physiological functions within virtually all cells [4], secretory PLA_2 _(sPLA_2_) enzymes are known to be active during inflammation, and thus have been an attractive target for anti-inflammatory drug development [3]. sPLA_2 _enzymes also have agonistic activity at the M-type receptor, through which they can promote inflammation via degranulation of mast cells, cytokine release or secretion of elastase, an activator of the complement cascade extrinsic pathway [5-8]. sPLA_2 _enzyme concentrations have been found to be elevated in the synovial fluid of patients with RA [2,9]. Correlations have also been found between serum levels of sPLA_2 _and clinical markers of disease such as the number of active and effused joints, erythrocyte sedimentation rate, Lansbury index, elevated platelet count, and low hemoglobin in RA patients [10,11]. Arthritic joints have also been shown to have high expression of sPLA_2 _group IIa within the synovial lining, while sPLA_2 _IIa expression in healthy joints is virtually absent [12]. Furthermore, intra-articular injections of human recombinant sPLA_2 _caused acute inflammatory arthritic-like symptoms in rats [13] and rabbits [14], although transgenic mice over-expressing human sPLA_2 _did not spontaneously develop arthritis [15,16].

Researchers from Eli Lilly performed a phase I clinical trial using an inhibitor of sPLA_2 _group IIa (LY315920) given intravenously to patients with active RA, which provided significant improvement in swollen and tender joints after three days [17]. Following this, a larger scale Phase II trial was conducted to evaluate the oral efficacy of LY333013, a methyl ester prodrug of LY315920. The results from this trial indicated that although there were significant dose-response related improvements after one week of treatment, there was no significant effect following four and eight weeks of treatment [17]. Potential explanations for this failure include the lack of sufficient inhibitor concentration in the synovial fluid to inhibit local joint sPLA_2_, and that all patients were already receiving disease-modifying anti-arthritic drug therapy throughout the trial [17,18]. Therefore, there is still a need to establish whether there may be a pathogenic role of sPLA_2 _enzymes in RA.

We have previously reported that a synthetic small molecule inhibitor of group IIa sPLA_2 _(sPLA_2_I; 5-(4-benzyloxyphenyl)-4S-(7-phenylheptanoylamino)-pentanoic acid) is orally active and has therapeutic efficacy in rat models of intestinal ischemia-reperfusion injury [19] and inflammatory bowel disease [20]. There has also been evidence of efficacy with this compound in a small, preliminary investigation in adjuvant-induced arthritic rats [21]. To evaluate this finding, the present study reports a full investigation of the potential of this agent to prevent and reverse signs of inflammatory disease in the rat antigen-induced arthritis model. In addition, we compared the *in vivo *activity of this sPLA_2_I to the conventional anti-arthritic agents, infliximab, leflunomide and prednisolone. In this rodent model of RA, we found that the sPLA_2_I reduced all measured markers of arthritis pathology and was more effective than conventional anti-arthritic treatments.

## Materials and methods

### Animals

Pathogen-free female Wistar rats weighing 225 to 275 g were used in this study and housed in cages with 12-hour light/dark cycles at 23°C, and 60% humidity. All animal experimentation conducted in this study was performed in accordance with National Health and Medical Research Council of Australia guidelines and with approval from the ethics committee of the University of Queensland.

### Model of antigen-induced monoarticular arthritis

Rats were sensitised with methylated bovine serum albumin (mBSA; Sigma Aldrich, St Louis, Missouri, USA)) in 0.5 ml Freund's complete adjuvant (Sigma, USA) as previously described [22,23]. Injections were administered subcutaneously in the rat's flanks, one at three weeks (Day -21) and the other at two weeks (Day -14) prior to arthritis induction (Day 0). At Day 0 animals were anaesthetised with ketamine (80 mg/kg, i.p.) and xylazine (12 mg/kg, i.p.). Arthritis was then induced in the right knee of each animal by aseptically injecting 0.5 mg of mBSA dissolved in 50 μL of saline into the joint space, while 50 μL of saline was injected into the left knee as a control.

The widths of both left and right knee joints were measured with a vernier caliper at regular intervals before arthritis induction and daily throughout the 14-day experiment. Following the induction of arthritis, gait impairment was assessed semi-quantitatively in each animal, by an investigator blinded to treatment groups. Animals were scored using a five-point scale (0 to 4) according to set criteria as previously described [23].

At the completion of the study, the animals were anaesthetised with intraperitoneal zolazapam (50 mg/kg) and xylazine (12 mg/kg). Blood was obtained from the inferior vena cava for the collection of serum and both left and right knee joints from animals were then dissected and fixed in 10% buffered formalin. Tissues were decalcified in a saturated EDTA solution for 21 days and embedded in wax. Sections (5 μm) were stained with hematoxylin and eosin and examined by a trained observer who was blinded to the treatments. Each tissue was scored on the degree of joint damage as previously described [23]. Scoring was based on a scale from 0 to 8. Scores of 0 were assigned to normal joints with no detectable abnormalities while scores increasing from 1 to 8 were reserved for the graded appearance of synovial cell proliferation, fibrosis, inflammatory cell infiltration, haemorrhage and cartilage destruction [23]. These parameters were chosen as this model of antigen-induced monoarticular arthritis produces a local inflammatory response in the affected knee and minimal bone and cartilage erosion, as has been previously described [22,23].

### TNFα measurement

Serum samples were assayed for TNFα concentrations using an enzyme-linked immunosorbent assay (ELISA) kit (BD Pharmingen, Franklin Lakes, New Jersey, USA). Samples were diluted 1:10 before assay was performed according to the manufacturer's instructions.

### Drug preparation

The group IIa sPLA_2_I (5-(4-benzyloxyphenyl)-4S-(7-phenylheptanoylamino)-pentanoic acid), was synthesised as previously described [20,21]. Leflunomide and prednisolone were sourced from Sigma (USA). These compounds were dissolved in olive oil to a final volume of 200 μL and administered by oral gavage. The TNFα inhibitor, infliximab (Remicade; Schering-Plough, Kenilworth, New Jersey, USA) was dissolved in saline and administered to rats via a single i.v. injection [24,25].

### Treatment groups

Two separate experimental trials were conducted to examine the effects of the therapeutics at preventing and also reversing disease. In the first "prevention" trial, rats (*n *= 10 per group) were orally dosed with the sPLA_2_I at either 1 or 5 mg/kg beginning two days prior to the induction of arthritis (Day -2), and then daily throughout. An arthritic control group received oral vehicle (olive oil) doses only.

In the second "reversal" trial, rats (*n *= 7 to 12 per group) were treated with compounds two days following the induction of arthritis (Day +2), once significant signs of arthritis were already apparent. The treatment groups were as follows: A. sPLA_2_I (5 mg/kg; *n *= 7); B. sPLA_2_I (10 mg/kg; *n *= 7); C. leflunomide (10 mg/kg; *n *= 10); D. infliximab (3 mg/kg; *n *= 8); and E. prednisolone (1 mg/kg; *n *= 10). Dosages were determined from available literature (prednisolone: [24,26,27], leflunomide: [28], infliximab: [24]). All treatment groups were orally dosed daily throughout the experimental period, except for infliximab, which was administered once, as an i.v. injection on Day +2. The arthritic control group (*n *= 12) was dosed with the vehicle only. Another group of rats (sham-operated rats; *n *= 8) did not have arthritis induced, and were dosed with the vehicle only to determine increases in knee size and weight gain due solely to growth.

### Data and statistical analysis

Where indicated, values are expressed as mean ± the standard error of the mean (SEM). Histopathological scores are presented as individual scores, with median. Data were analysed using GraphPad Prism 5 software (GraphPad Software Inc., La Jolla, California, USA). Statistical comparisons were made using a one-way ANOVA with a Dunnett post-test, or a Mann-Whitney U test for histopathology scores. Results were considered significant when *P *< 0.05.

## Results

### Trial 1 (Prevention): The effect of sPLA_2_I pre-treatment on joint swelling and gait impairment

**Figure 1:**
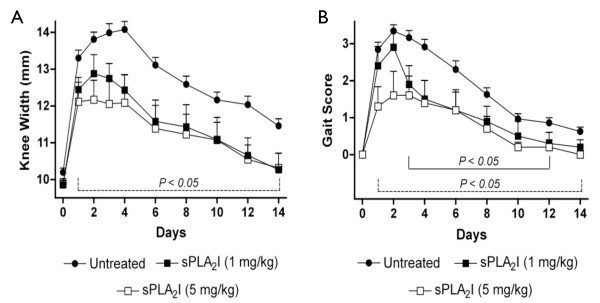
**Effect of sPLA2I pre-treatment on joint swelling and lameness**. Right knee joints of all sensitised rats were injected with antigen on Day 0. Knee widths (**A**) and gait scores (**B**) were recorded for each rat over 14 days. Treatment was commenced two days prior to arthritis induction and daily throughout. (A) Both oral doses of the sPLA2I resulted in a significant reduction of knee swelling throughout the study period. (B) Both oral doses of the sPLA2I resulted in a significant reduction of gait scores at various time points over the study period. Each data point represents the mean ± SEM. Lines indicate the period of significance (*P *< 0.05) between arthritis control and (A) both treatment groups; (B) dotted line - sPLA2I (5 mg/kg); solid line - sPLA2I (1 mg/kg).

Saline-injected left knees of rats did not significantly change in width from pre-injection values during the course of each experiment (data not shown). Injection of mBSA at Day 0 to the right knees of rats resulted in a significant increase in knee widths over the 14-day study period (Figure 1A). Rats treated orally with either dose of sPLA_2_I (1 or 5 mg/kg) two days prior to the injection of mBSA (Day -2), and daily throughout, had significantly reduced knee widths throughout the entire study (Days 1 to 14) compared to untreated, arthritis control rats (*P *< 0.05; Figure 1A).

The induction of arthritis in rats also resulted in considerable gait impairment which was measured via a gait score (Figure 1B). Rats pre-treated orally with the lower dose of sPLA_2_I (1 mg/kg) had significantly reduced knee widths from Days 3 to 12 compared to untreated, arthritis control rats (*P *< 0.05; Figure 1B). Rats pre-treated orally with the higher dose of sPLA_2_I (5 mg/kg) had significantly reduced knee widths throughout the entire study period (Days 1 to 14) compared to untreated, arthritis control rats (*P *< 0.05; Figure 1B).

Untreated, arthritis control rats also lost weight over the course of the study (Day 14 body weight: 3.0 ± 1.6 grams lost from Day 0 body weight). In contrast, rats pre-treated with the sPLA_2_I (1 or 5 mg/kg) gained significant weight at the completion of the study (Day 14 body weight: 8.0 ± 2.2 and 6.5 ± 3.1 grams gained respectively from Day 0 body weight; *P *< 0.05 compared to untreated, arthritis control rats; data not shown)

### Trial 1 (Prevention): Effect of sPLA_2_I pre-treatment on joint histopathology

**Figure 2:**
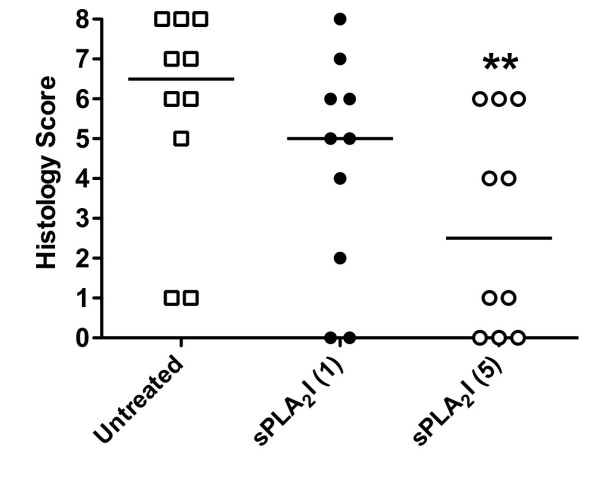
**Effect of sPLA2I pre-treatment on histopathology of joint**. At the completion of the 14-day pre-treatment study all arthritic knee joints were removed and prepared for histological evaluation. Knee sections were scored (0 to 8) for the degree of cellular infiltration, synovial hyperplasia, cartilage damage and bone erosions. Only rats orally treated with the sPLA2I at 5 mg/kg/day showed a significant reduction in histopathology scores. Each data point represents a specific score with the median shown. ** indicates significant difference (*P *< 0.01) between arthritis controls and treatment group using the Mann-Whitney U test.

At the completion of the study, knee joints were examined histologically and scored on the degree of damage by an experienced observer, blinded to the treatment groups (Figure 2). All saline-injected, left knees of rats had no observable pathology (data not shown). The induction of arthritis to the right knees of rats resulted in distinct cellular infiltration, which was predominantly neutrophils, with synovial cell proliferation and hyperplasia. Cartilage erosions, however, were minimal. Rats orally pre-treated with the sPLA_2_I at the higher dose (5 mg/kg) had significantly reduced joint histopathology scores compared to untreated, arthritis control rats (*P *< 0.05; Figure 2). In contrast, rats pre-treated with the lower dose of the sPLA_2_I (1 mg/kg) did not show a significant reduction in joint histopathology scores (*P *> 0.05; Figure 2).

### Trial 1 (Prevention): Effect of arthritis induction on circulating cytokine levels

At Day 14, serum from diseased- and sham-treated rats showed no significant difference in TNFα concentrations (0.41 ± 0.02 and 0.37 ± 0.01 respectively; *P *= 0.69). These data are in accordance with previous research that describes this model as a local, rather than systemic, model of RA [22]. Therefore, it was decided not to apply this measurement to future experiments.

### Trial 2 (Reversal): Effect of drug post-treatment on joint swelling and gait impairment

**Figure 3:**
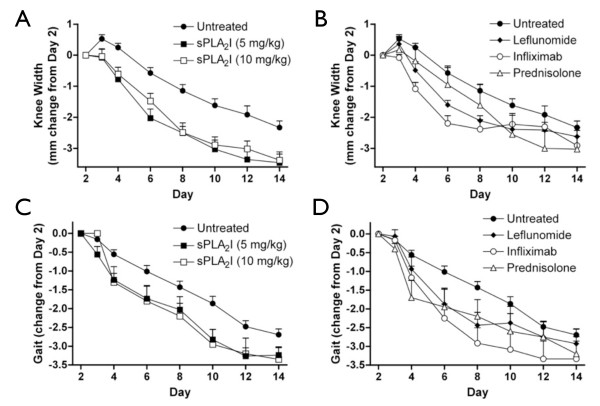
**Effect of post-treatment on joint swelling and lameness**. Right knee joints of all sensitised rats were injected with antigen on Day 0. Treatment was commenced two days following arthritis induction. Knee widths (**A, B**) and gait scores (**C, D**) were then recorded for each rat and are displayed as a change in these parameters from point of treatment (Day 2). Treatment with the sPLA2I at both doses (5 and 10 mg/kg) resulted in a significant reduction in joint swelling (A) (*P *< 0.05; Days 3 to 14) and gait scores (C) (*P *< 0.05; Days 4, 10, 12). Treatment with the comparator drugs (leflunomide, infliximab and prednisolone) also resulted in significant reductions (*P *< 0.05) in joint swelling (B) and gait scores (D) although to a lesser extent than the sPLA2I (knee widths: leflunomide (Days 4 to 8), infliximab (Days 3 to 8), prednisolone (Day 10); gait scores: leflunomide (Days 6, 8), infliximab (Days 6 to 10),to prednisolone (Day 4)). Each data point represents the mean ± SEM.

Treating animals two days following the induction of arthritis (Day +2) and daily throughout the study with the sPLA_2_I (5 and 10 mg/kg) significantly ameliorated joint swelling throughout the entire study period following treatment (*P *< 0.05, Days 3 to 14; Figure 3A). Animals treated from Day +2 with the various comparator drugs: leflunomide (10 mg/kg), infliximab (3 mg/kg), and prednisolone (1 mg/kg) also had significant reductions in joint swelling, although for a reduced time period than the sPLA_2_I (*P *< 0.05, leflunomide (Days 4 to 8), infliximab (Days 3 to 8), prednisolone (Day 10); Figure 3B).

Gait scores significantly improved following treatment with either dose of the sPLA_2_I (5 or 10 mg/kg) from Day +2 (*P *< 0.05, Days 4, 10, 12; Figure 3C). Animals treated with the various comparator drugs from Day +2 also had significant reductions in gait scores, although again for a shorter time period than the sPLA_2_I (*P *< 0.05, leflunomide (Days 6, 8), infliximab (Days 6 to 10), prednisolone (Day 4); Figure 3D).

**Figure 4:**
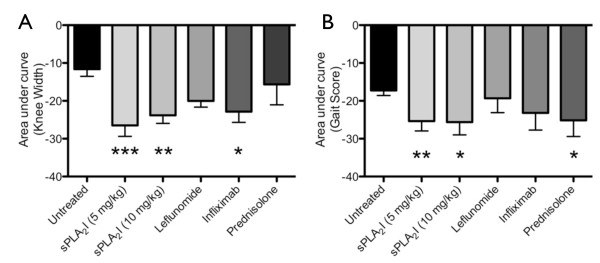
**Effect of post-treatment on joint swelling and lameness throughout the study period**. Right knee joints of all sensitised rats were injected with antigen on Day 0. Treatment was commenced two days following arthritis induction. Representative reduction in knee width (**A**) and gait score (**B**) throughout the trial period (Days 0 to 14) was attained by calculating the average area under the curve of data displayed in Figure 3. sPLA2I treatment at 5 or 10 mg/kg achieved a significant reduction in both joint swelling (*P *< 0.001 and *P *< 0.01 respectively) and lameness (*P *< 0.01 and *P *< 0.05 respectively) over the trial period (A, B). Infliximab significantly reduced joint swelling (*P *< 0.05) without effecting lameness (A), and prednisolone significantly reduced lameness (*P *< 0.05) without effecting swelling (B). Each column represents mean ± SEM.

Joint swelling and gait scores were assessed over the entire trial period by comparing area under the curves from Figure 3. Overall, throughout the trial period, only sPLA_2_I (5 or 10 mg/kg) showed a significant difference in both knee width and gait score (5 mg/kg: *P *< 0.001 and 0.01; 10 mg/kg *P *< 0.01 and 0.05; Figure 4). Infliximab demonstrated an overall significant decrease in knee width (*P *< 0.05) and prednisolone demonstrated an overall decrease in gait score (*P *< 0.05); leflunomide did not reach significance for either parameter (*P *> 0.05; Figure 4).

### Trial 2 (Reversal): Effect of drug post-treatment on body weight loss

**Figure 5:**
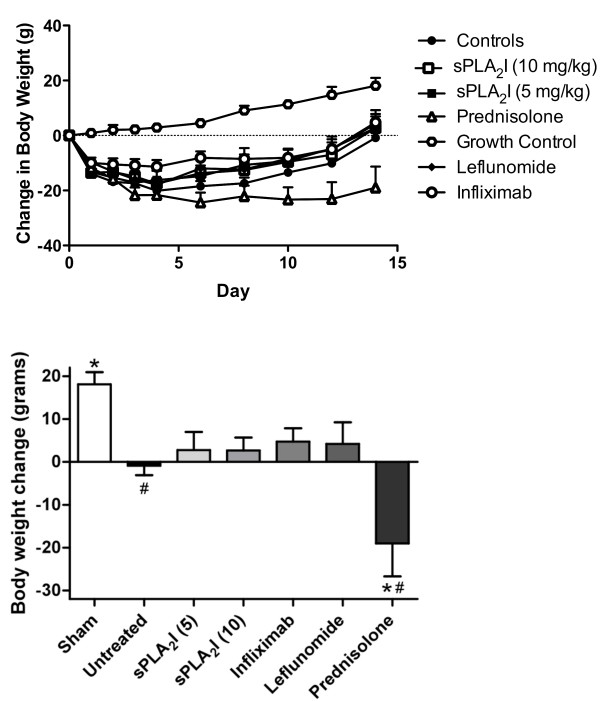
**Effect of post-treatment on body weight**. Right knee joints of all sensitised rats were injected with antigen on Day 0, and the change in body weight from Day 0 recorded every 48 hours for 14 days. Treatment was commenced two days following arthritis induction. The panels show: (**A) **Body weight over time for all treatment groups. **(B) **Change in body weight from baseline values. Arthritis control rats lost weight after 14 days, while all treatment groups gained weight, except for prednisolone-treated rats which lost considerable weight. Each data point represents the mean ± SEM. (*P *< 0.01). Significant differences from sham-operated rats (#) and arthritis controls (*) are indicated (*P *< 0.01).

Rats were weighed throughout the experiment, with final day (Day +14) weights expressed as a change in weight from arthritis induction (Day 0) and compared between the various groups (Figure 5). Untreated, arthritis control rats had a total weight loss over the 14 days, and had significantly reduced weight compared to un-diseased, sham-operated rats (*P *< 0.05; Figure 5). All drug treatments, with the exception of prednisolone, resulted in an increase in weight after 14 days, though none were significantly increased from untreated, arthritic control rats (*P *> 0.05; Figure 5. Prednisolone-treated rats showed considerable weight loss over the study period, and after 14 days had significantly reduced weight compared to untreated, arthritic control rats (*P *< 0.05; Figure 5).

### Trial 2 (Reversal): Effect of drug post-treatment on joint histopathology

**Figure 6:**
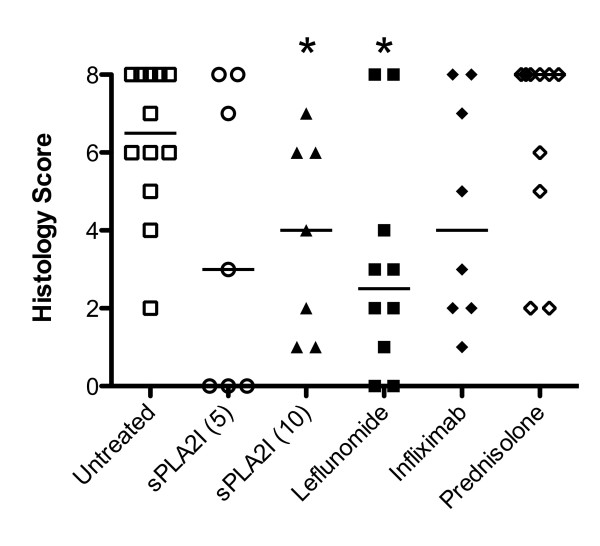
**Effect of post-treatment on histopathology of joint**. At the completion of the 14-day post-treatment study all arthritic knee joints were removed and prepared for histological evaluation. Knee sections were scored (0 to 8) for the degree of cellular infiltration, synovial hyperplasia, cartilage damage and bone erosions. Rats treated with the sPLA_2_I (5 mg/kg) and infliximab showed reduced median scores compared to controls, though no significance was obtained. Treatment with sPLA_2_I (10 mg/kg) and leflunomide exhibited significantly reduced histopathology scores (*P *< 0.05, Mann-Whitney U test). Each data point represents the specific score with the median shown.

Histological analysis and scoring of diseased joints for untreated, arthritic control rats showed a similar degree of pathology compared to the same group in the first trial (Figure 6). Similarly, only rats treated with the higher dose of sPLA_2_I (10 mg/kg) showed a significant reduction in histopathological scores (*P *< 0.05; Figure 6), although a reduction in median histopathological scores was seen with the lower sPLA_2_I (5 mg/kg). Leflunomide treatment also resulted in a significant improvement in histopathology scores (*P *< 0.05; Figure 6). Rats treated with infliximab showed a non-significant reduction median scores, (*P *> 0.05; Figure 6) and rats treated with prednisolone showed a clear lack of benefit, with no significant reductions in these scores (*P *> 0.05; Figure 6).

## Discussion

This study is the first investigation of sPLA_2 _IIa inhibition in the antigen-induced arthritis model of RA. We have previously demonstrated the usefulness of this model in establishing the efficacy of other experimental compounds and conventional anti-inflammatory drugs [23]. In the present study we have compared the efficacy of sPLA_2 _group IIa enzyme inhibition, using an orally-active and specific small molecule sPLA_2_I, with currently used anti-arthritic drugs in reducing antigen-induced arthritic pathology. We demonstrate that inhibition of sPLA_2 _IIa alleviates the clinical signs and pathological changes associated with RA, with a greater reliability than some conventional anti-rheumatoid therapies.

sPLA_2 _IIa is a secretory enzyme that converts arachidonic acid (AA)-containing phospholipids to free AA, and has been shown to be highly expressed in affected joint tissues in patients with RA [12]. sPLA_2 _IIa forms the apex of an autacoids cascade in the synovium of arthritic joints. The heparin binding domain of sPLA_2 _localises to lipid rafts, bringing the enzyme into close proximity to downstream mediators of this cascade, such as cycloxygenase and lipoxygenase [29]. Additionally, sPLA_2 _IIa is a ligand for the M-type receptor located on inflammatory cells [5]. Signalling through the M-type receptor in mast cells results in degranulation [30]; in neutrophils it mediates an increase in cPLA_2 _[29]; and in monocytes induces exocytosis of cytokines, including TNFα [31]. Current RA therapies, such as the TNFα inhibitor, infliximab, or the NSAID, ibuprofen, target the mediators downstream of sPLA_2 _IIa. Specific inhibition of sPLA_2 _IIa may, therefore, be a valid target to develop novel disease modifying anti-rheumatic drugs (DMARDs) which are more efficacious than existing therapies, given high concentrations of sPLA_2 _IIa in arthritic joints [23]. This study confirms this hypothesis demonstrating that an orally-active sPLA_2_I in a rat model of RA provides significant benefits over inhibition of downstream mediators of inflammation currently used as standard therapies in the treatment of RA.

In this study, we used a potent and orally active inhibitor of group IIa sPLA2 enzymes (sPLA_2_I) [20,21]. Oral administration of this drug to rats, prior to the induction of arthritis and daily throughout the trial, was found to be effective at reducing joint swelling and gait score when administered at both 1 and 5 mg/kg/day. However, sPLA_2_I at the lower 1 mg/kg dose failed to reduce the disease progression as demonstrated by histopathology, when compared to untreated controls. Therefore, doses of 5 and 10 mg/kg/day were used to examine efficacy of reversing established arthritic damage. It is likely that the effect of sPLA_2_I demonstrated in the prevention trials is due to action on the effector, rather than induction-phase of the immune response as rats were pre-sensitised to the antigen at -21 and -14 days. However, the design of this study did not allow us to discriminate between the action of the drug on both phases. In the reversal therapeutic trial, sPLA_2_I was compared to conventional arthritis treatments infliximab, leflunomide and prednisolone. Rats were treated from Day 2, as this is near the maximal response in knee swelling and gait scores as seen in our first experimental trial. A separate group of rats euthanased at Day 2 showed a significant degree of histopathological damage (data not shown) validating the choice to initiate treatment at this time point.

Both 5 and 10 mg/kg/day sPLA_2_I significantly reduced both gait score and joint swelling over the course of the study in the reversal trial. Of the conventional treatments, although all were able to demonstrate a significant benefit at certain individual time points, only infliximab reduced inflammation (knee width) and prednisolone reduced pain (gait score) with overall statistical significance. Additionally, treatment with infliximab or prednisolone showed no significant reduction in histopathology score, but both leflunomide and sPLA_2_I (10 mg/kg/day) were effective in reducing the joint histopathology to a significant degree. Overall, sPLA_2_I alleviated all aspects of RA pathology with a greater reliability than any of the conventional therapeutics in this study.

The success of targeted sPLA_2 _IIa inhibition with the sPLA_2_I used in this study could be attributed to the actions of sPLA_2 _IIa upstream of many of the targets of the conventional therapies. For instance, the binding of sPLA_2 _IIa to the M-type receptors has been shown to cause TNFα, IL6 and IL12 release from monocytes [32]; in this way sPLA_2_I has clinical similarities to those of infliximab, a TNFα inhibitor. Additionally, sPLA_2 _inhibition prevents mast cell degranulation by inhibiting sPLA_2 _IIa interaction with the M-type receptor [6] whereas leflunomide induces mast cell apoptosis [33]. Mast cells have been postulated as the link between the antigen-antibody complex triggered inflammation and sustained, chronic RA, as mast cell deficient mice are resistant to the development of RA in an arthritogeneic serum model [29]. This mechanism could explain the success of early leflunomide intervention in clinical trials, and the significant reduction in histopathology score, by both sPLA_2_I and leflunomide, in this study [34,35]. We have previously demonstrated that another commonly used anti-arthritic agent, ibuprofen, was unable to reduce the degree of histological damage in the same model, despite providing a therapeutic effect for joint swelling and gait scoring [23]. This is in contrast to leflunomide, which was able to reduce histopathology without providing an overall significant benefit to gait score and joint swelling after Day 8. Inhibition of sPLA_2 _IIa, however, caused significant reductions in gait score, joint swelling and histopathology. This is evidence that each of these facets of RA is mediated by separate, but communicating, mechanisms. sPLA_2 _inhibition reduces prostaglandin synthesis by COX, through reducing the concentration of free AA, whilst ibuprofen is a direct inhibitor of COX. This inhibition of prostaglandin synthesis, which both reduces inflammation and inhibits pain, may account for the significant difference seen in gait scores/joint swelling with both these drugs. Inhibition of sPLA_2 _IIa also reduces mast cell degranulation and neutrophil infiltration by preventing binding to M-type receptors [33]. This attenuation of immune cell function may mimic the mast cell-specific repression of leflunomide, which causes cell cycle arrest and mast cell apoptosis, in alleviating joint histopathology [33]. The multiple actions of sPLA_2 _IIa in arthritis, and its absence in the synovium of healthy joints, are likely the reason for its success in this study when compared to conventional therapeutics. In addition, conventional therapeutics, with the exception of prednisolone, target single downstream mediators of sPLA_2 _IIa actions.

**Figure 7:**
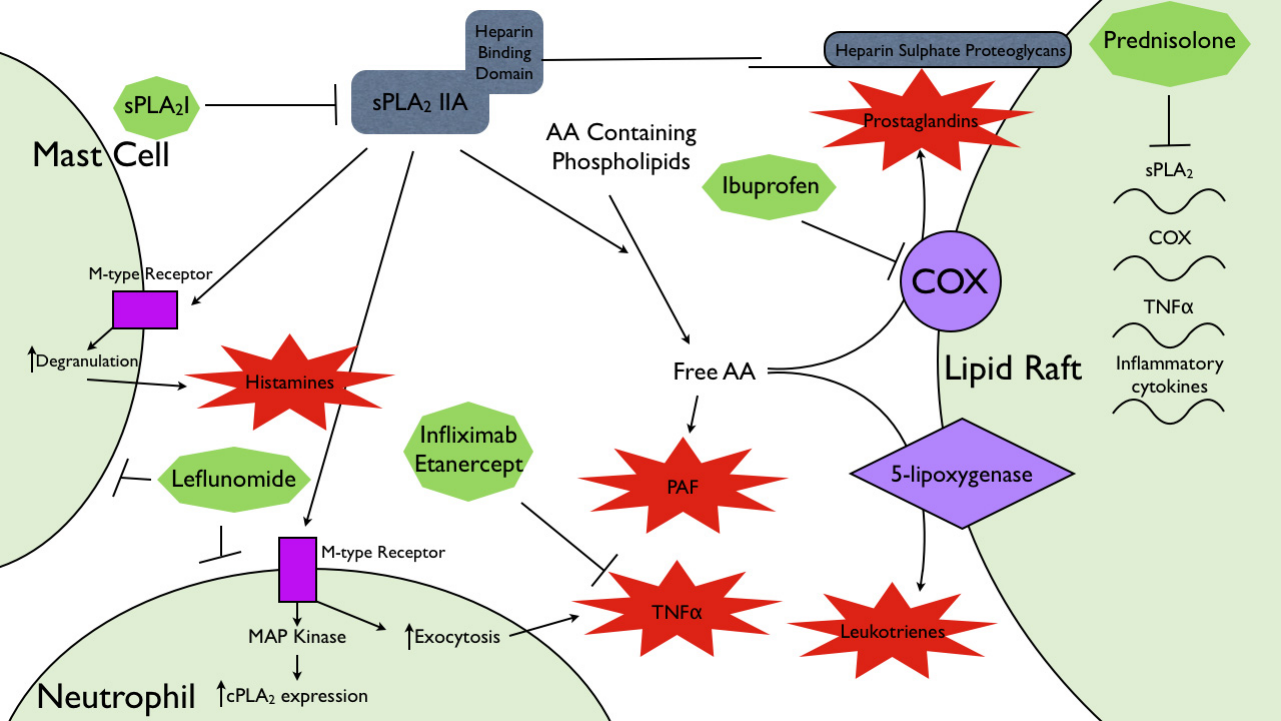
**Simplified hypothetical model demonstrating the sites of action of anti-arthritic therapeutics used in this study**. sPLA_2 _IIa provides the substrate for the production of the inflammatory mediators; platelet-activating factor (PAF), prostaglandins and leukotrienes. Additionally, sPLA_2 _IIa is a ligand for the M-type receptor: sPLA_2 _IIa agonist activity causes mast cell degranulation and activation of neutrophils. Ibuprofen, leflunomide and infliximab all act on inflammatory mediators downstream of sPLA_2 _IIa actions. Prednisolone globally alters inflammatory gene transcription. AA, arachidonic acid; cPLA_2_, cytosolic phospholipase A_2; _PAF, platelet activating factor; MAP kinase, mitogen activated protein kinase; M-type receptor, muscle type receptor; sPLA_2 _IIa, secretory phospholipase A_2 _group IIa; TNFα, tumour necrosis factor alpha.

Specific inhibition of sPLA_2 _enzymes has an advantage over conventional RA therapeutics, in that it targets multiple possible pathways of RA pathogenesis (Figure 7), without affecting normally occurring biological processes. There have been no demonstrated side effects of sPLA_2 _IIa inhibition in animal models of disease and the phase II clinical trial of an sPLA_2_I in humans provided evidence of some liver toxicity only at the highest doses (1,000 mg/day) [17]. Conversely, all conventional therapeutics in this study failed to show significant benefit to every pathology measurement of the antigen-induced arthritis model. In addition, many of the conventional therapeutics have off-target effects that mitigate the benefits provided. Leflunomide and infliximab have been shown to inhibit osteogenic cell proliferation [36]; because of this, both treatments pose additional risks for post-menopausal women. Leflunomide and prednisolone actively suppress the immune system; leflunomide induces the apoptosis of several types of immune cells [33,37,38], and prednisolone induces cell cycle arrest, thereby inhibiting cellular proliferation. This study also confirms a catabolic effect of daily prednisolone administration as has been previously shown [24] and, although prednisolone can act as an inhibitor of sPLA_2 _IIa transcription [39], the use of a low-dose steroid in a chronic condition can potentiate morbidity for the patient [40-42].

Previously, the efficacy of sPLA_2 _IIa inhibition has been tested in phase II clinical trials as an adjunct to DMARD therapy without success [17]. The administration of DMARDS in conjunction with the sPLA_2_I may have masked any benefits provided by sPLA_2_I. Here we show, for the first time, that sPLA_2 _IIa inhibition has the potential to be a useful monotherapy for the treatment of RA, and may be a more effective chronic therapy than the conventional RA therapeutics.

## Conclusions

We have previously shown the sPLA_2_I used in this study to be an orally-active, highly selective drug for the treatment of intestinal ischemia reperfusion injuries [19] and inflammatory bowel disease [20] in rat models, and now demonstrate its efficacy in a model of RA. Inhibition of sPLA_2 _IIa using a chemically different sPLA_2 _enzyme inhibitor, has previously been trialed in human RA. In this clinical trial, the sPLA_2 _inhibitor was administered as an adjunct therapy to DMARD and glucocorticoid therapy, which may have masked any benefits to patients [17]. By directly comparing sPLA_2 _inhibition to conventional therapies in a rodent model of antigen-induced arthritis, we have provided a rationale and evidence for the use of sPLA_2_I as a replacement for DMARD/glucocorticoid therapy in future clinical trials.

## Abbreviations

AA: arachidonic acid; DMARD: disease modifying anti-rheumatic drug; mBSA: methylated standard error of the mean; PAF: platelet activating factor; sPLA_2_: secretory phospholipase A_2_; sPLA_2_I: secretory phospholipase A_2 _inhibitor; TNFα: tumour necrosis factor alpha.

## Competing interests

The authors declare that they have no competing interests.

## Authors' contributions

TW, IS and ST participated in the design of the study and interpretation of data. TW, JC, BR and LC participated in the acquisition and analysis of the data. IS performed the histopathological analysis and scoring of the knee joints. TW and LC drafted the manuscript and ST and IS assisted in editing the manuscript. All authors read and approved the final manuscript.
